# Stem cell collection in unmanipulated HLA-haploidentical/mismatched related transplantation with combined granulocyte-colony stimulating factor-mobilised blood and bone marrow for patients with haematologic malignancies: the impact of donor characteristics and procedural settings

**DOI:** 10.1111/j.1365-3148.2010.00990.x

**Published:** 2010-06

**Authors:** C Zhang, X-H Chen, X Zhang, L Gao, L Gao, P-Y Kong, X-G Peng, A-H Sun, Y Gong, D-F Zeng, Q-Y Wang

**Affiliations:** Department of Hematology, Xinqiao Hospital, The Third Military Medical UniversityChongqing 400037, People's Republic of China

**Keywords:** granulocyte-colony stimulating factor, HLA-haploidentical/mismatched related donors, leukaemia, mobilisation, transplantation

## Abstract

Unmanipulated haploidentical/mismatched related transplantation with combined granulocyte-colony stimulating factor-mobilised peripheral blood stem cells (G-PBSCs) and granulocyte-colony stimulating factor-mobilised bone marrow (G-BM) has been developed as an alternative transplantation strategy for patients with haematologic malignancies. However, little information is available about the factors predicting the outcome of peripheral blood stem cell (PBSC) collection and bone marrow (BM) harvest in this transplantation. The effects of donor characteristics and procedure factors on CD34^+^ cell yield were investigated. A total of 104 related healthy donors received granulocyte-colony stimulating factor (G-CSF) followed by PBSC collection and BM harvest. Male donors had significantly higher yields compared with female donors. In multiple regression analysis for peripheral blood collection, age and flow rate were negatively correlated with cell yield, whereas body mass index, pre-aphaeresis white blood cell (WBC) and circulating immature cell (CIC) counts were positively correlated with cell yields. For BM harvest, age was negatively correlated with cell yields, whereas pre-BM collection CIC counts were positively correlated with cell yield. All donors achieved the final product of ≥6 ×10^6^ kg^−1^ recipient body weight. This transplantation strategy has been shown to be a feasible approach with acceptable outcomes in stem cell collection for patients who received HLA-haploidentical/mismatched transplantation with combined G-PBSCs and G-BM. In donors with multiple high-risk characteristics for poor aphaeresis CD34^+^ cell yield, BM was an alternative source.

HLA-haploidentical/mismatched related haematopoietic stem cell transplantation (HSCT) has been limited by delayed engraftment, high risk of severe graft-versus-host disease (GVHD), graft rejection and life-threatening infections (Schattenberg *et al.*, 2005). To overcome these problems, unmanipulated HLA-haploidentical/ mismatched related HSCT was performed with combined granulocyte-colony stimulating factor-mobilised peripheral blood stem cells (G-PBSCs) and granulocyte-colony stimulating factor-mobilised bone marrow (G-BM) (Chen *et al.*, 2009). With G-BM and G-PBSCs as source of grafts, more haematopoietic stem cells may be harvested than via use of bone marrow (BM) or peripheral blood (PB) alone. However, granulocyte-colony stimulating factor (G-CSF) markedly increased donor-derived mesenchymal stromal cells in the BM, which can modulate GVHD pathophysiology and graft-versus-leukaemia (GVL) and possibly accelerate haematopoietic recovery (Sorror *et al.*, 2004; Ball *et al.*, 2007; Tatsumi *et al.*, 2008).

Although the number of mismatched allogeneic transplants has increased steadily over the past few decades, the unmanipulated HLA-haploidentical/mismatched HSCT from related donors was still a small portion. Identifying factors associated with better quality and greater quantity of stem cells may provide an opportunity to enhance patient recovery while ensuring a safe process for stem cell donation for the HLA-haploidentical/mismatched related donors. In order to gain insight into the factors that may affect the quantity of stem cell collection for leukaemic patients who undergo unmanipulated HLA-haploidentical/mismatched related HSCT with combined G-BM and G-PBSCs, we retrospectively analysed the effects of donor characteristics and procedure factors on CD34^+^ cell yield from a single centre in China.

## DONORS AND METHODS

### Donors

Between 2005 and December 2008, a total of 104 healthy related volunteers underwent peripheral blood stem cell (PBSC) and BM harvest in our institution. Donor assessments were in accordance with the standards of donor evaluation for allogeneic transplantations (Sierra *et al.*, 1997). Informed consent was obtained from each donor. HLA-A, HLA-B and HLA-DR typing were performed by serology for all donor–recipient pairs. The degree of the locus mismatch conforms to the China consensus (Huang *et al.*, 2004) on immunogenetic donor search for transplantation full mismatch. Tables 1 and 2 show the characteristics and procedural variables in the cohort, respectively.

**Table 2:** Procedural variables in related donors (*n* = 104)

Variable	
G-CSF dose, µg kg^−1^	9·2 (6·8–10·9)
No. of aphaereses per mobilisation cycle	1 (1–1)
Actual flow rate of aphaereses, mL min^−1^	53·7 (41·7–66·7)
Total processed blood volume, L	9·7 (5–12)
Processed blood volume per donor BW, mL kg^−1^	160·6 (81·6–227·3)
Pre-aphaeresis WBC count (×10^9^ L^−1^)	41·9 (22·3–65·6)
Pre-aphaeresis HGB (g L^−1^)	120·7 (97–143)
Pre-aphaeresis platelet count (×10^9^ L^−1^)	149·5 (78–237)
Pre-aphaeresis CICs count (×10^9^ L^−1^)	9·7 (4·5–23)
Total CD34^+^ cell count for PB (×10^6^)	250·9 (47·6–435)
CD34^+^ cells per donor BW for PB (×10^6^ kg^−1^)	4·0 (0·9–7·4)
CD34^+^ cells per recipient BW for PB (×10^6^ kg^−1^)	4·8 (1·2–12·7)
CD34^+^ cell yield, 10^6^ L^−1^ of processed blood	26·1 (7·4–48·3)
No. of BM collection per mobilisation cycle	1 (1–1)
Total processed BM volume, L	0·86 (0·4–1·4)
Processed BM volume per donor BW, mL kg^−1^	14·3 (6·8–26·4)
Pre-BM collection WBC count (×10^9^ L^−1^)	45·0 (32·0–61·9)
Pre-BM collection HGB (g L^−1^)	112·0 (75·0–176·0)
Pre-BM collection platelet count (×10^9^ L^−1^)	107·5 (64·0–201·0)
Pre-BM collection CICs count (×10^9^ L^−1^)	9·4 (4·3–21·1)
Total CD34^+^ cell count for BM before management (×10^6^)	395·7 (102–1282)
CD34^+^ cells per donor BW for BM before management (×10^6^ kg^−1^)	6·8 (1·6–28·9)
CD34^+^ cells per recipient BW for BM before management (×10^6^ kg^−1^)	7·9 (2·1–25·4)
CD34^+^ cell yield, 10^6^ L^−1^ of processed BM before management	492·6 (130·2–1411·1)
Total CD34^+^ cell count for BM after management (×10^6^)	374·9 (88·7–1230·7)
CD34^+^ cells per donor BW after management (×10^6^ kg^−1^)	6·4 (1·6–25·7)
CD34^+^ cells per recipient BW after management (×10^6^ kg^−1^)	7·5 (2·1–23·2)
CD34^+^ cell yield, 10^6^ L^−1^ of processed BM after management	468·0 (118·3–1255·9)
Total collected CD34^+^ cell count (×10^6^)	625·8 (136·3–1506·3)
Total collected CD34^+^ cells per donor BW (×10^6^ kg^−1^)	10·6 (3·1–29·1)
Total collected CD34^+^ cells per recipient BW (×10^6^ kg^−1^)	12·3 (6·3–30·7)

Unless noted otherwise, data are presented as mean (range); BM, bone marrow; BW, body weight; CICs, circulating immature cells; G-CSF, granulocyte-colony stimulating factor; HGB, haemoglobin; PB, peripheral blood; WBC, white blood cell.

**Table 1:** Donors' characteristics in related donors (*n* = 104)

Variable	
No. of donors	104
Sex^1^	
Female	44 (42·3%)
Male	60 (57·7%)
Age, years (range)	39·0 (14–59)
0 to 18	8
19–40	56
>40	40
HLA-antigen class mismatched	
Class I	20
Class II	12
Class I and class II	72
No. of HLA-antigen mismatched	
1	24
2	12
3	68
No. and location of mismatched loci	
Only at HLA-A	8
Only at HLA-B	4
Only at HLA-DRB1	12
Mismatches at HLA-A, -B	8
Mismatches at HLA-B and -DRB1	4
Mismatches at HLA-A, -B and -DRB1	68
BMI, kg m^−2^	23·2 (15·6–31·6)

Unless noted otherwise, data are presented as mean (range); BMI, body mass index.

1 Data presented as number (%).

### PBSC mobilisation

The mobilisation of PBSCs was done according to our previous report (Chen *et al.*, 2009). Each donor received the G-CSF subcutaneously at a dose of 10 µg kg^−1^ body weight (BW) per day (given as a split dose twice daily) at approximately the same time every day. To avoid calculation errors, the actual dose was calculated at the time of the donor health examination.

### PBSC and BM collection

Leukaphaeresis was carried out on the fourth day after G-CSF mobilisation with the aid of an automated continuous flow blood cell separator (CS3000; Baxter, Deerfield, IL, USA) at a planned flow rate of 40–60 mL min^−1^. The actual flow rate was calculated by dividing the total processed blood volume (mL) by the processing time (min). Aphaeresis was performed once for all cases.

The donor BM was harvested at room temperature in the operation room on the fifth day after G-CSF treatment under epidural anaesthesia. The plasma and red blood cell (RBC) were excluded from the collected BM by hypothermic centrifugal machine and hydroxyethyl starch for the ABO minor and major mismatched donors, respectively. Both the plasma and RBC were separated for both the ABO minor and major mismatched donors. G-PBSCs and G-BM were transfused intravenously to the recipients just after the completion of the collection on days 1 and 2 of transplantation, respectively. Calcium was intravenously administered prophylactically during leukaphaeresis to reduce signs of hypocalcaemia. Daily counts and serum chemistry were obtained during G-CSF administration and in addition 30 days after the last administration of G-CSF. Donors complaining of bone pain received naproxen. The autologous RBC was prepared 10–14 days before aphaeresis and transfused during BM collection.

### CD34^+^ cell counts

PB cell counts before aphaeresis and BM harvest and PBSC and BM component counts were obtained using an automated cell counter (Model SE-9000; Sysmex Corporation, Kobe, Japan). White blood cells (WBCs), circulating immature cells (CICs), platelets and CD34^+^ cells were counted for analysis. CICs were defined as circulating myeloblasts, promyelocytes, myelocytes, metamyelocytes and erythroblasts detected by morphology in a PB smear; their counts were calculated by multiplying the percentage of the CICs with each donor's WBC count (Sierra *et al.*, 1997).

CD34^+^ content of collected products was quantitated by flow cytometric analysis as reported previously (Zhang *et al.*, 2008). Briefly, 100 µL of each sample, containing (5–10) × 10^5^ cells, was incubated for 10 min at room temperature with phycoerythrin [PE; (HPCA-2)]-conjugated anti-CD34^+^ monoclonal antibody (moAb), fluorescein isothiocyanate-conjugated anti-CD33 moAb and PerCP-conjugated anti-CD45 moAb (Becton Dickinson, Mountain View, CA, USA), whereas the control reagent contained a nucleic acid dye, PE-labelled IgG1 and PerCP-labelled anti-CD45 antibody. After red cell lysis with lysing solution, the CD34^+^ cell count was performed using a flow cytometer (FACScan, Becton Dickinson) and a ProCOUNT software package (BD) for each sample. At least 80 000 CD45 events and 1500 BD TruCOUNT tube beads were collected in each sample. Samples were analysed according to the gating strategy as described previously (Sierra *et al.*, 1997). Nucleic acid dye vs. side-scattering cytogram (SSC) and CD45 PerCP vs. SSC dot plots were used to exclude cellular debris and any event with bright CD45 or high SSC, which are not characteristic of progenitor cells. The CD34^+^ cell population was defined in the nucleic acid dye vs. the CD34^+^ PE of cells gated from previous additive regions. The absolute number of CD34^+^ cells in the sample was determined by dividing the number of CD34^+^ cellular events by the number of fluorescent bead events and then multiplying this by the bead concentration. Stem cell yield was defined as the number of the CD34^+^ cells kg^−1^ of recipient BW. A final CD34^+^ cell count of <2 × 10^6^ cells kg^−1^ (recipient BW) was considered a poor yield in our outcome analysis.

### Statistical evaluation

Descriptive statistics, including mean and range, were calculated, and age, body mass index (BMI) and aphaeresis and BM products for male and female donors and recipients were compared using the two-sample *t*-test and the χ^2^ test. BMI was defined as the individual's BW in kilograms divided by height squared in metres (kg m^−2^). The means of procedural variables, including actual flow rate, processed volume and CD34^+^ cell yield for PB and BM were compared using two-sample *t*-tests based on all data and by stratified gender group separately. Multiple regression was used to analyse the correlations among potential factors, including donor characteristics, procedural variables and CD34^+^ cell yields. The risk for poor CD34^+^ cell yields was evaluated using a logistic regression model. A test was considered statistically significant when the *P* value was under 0·05. All analyses were performed using the spss 13·0 statistical package (SPSS Inc, Chicago, IL).

## RESULTS

### Donor characteristics

The mean donor age was 39, with a predominance of males (57·7%). The mean G-CSF dose was 9·2 mg kg^−1^. It was the first stem cell donation and PBSC collection and BM harvest for all donors. The three loci of mismatched was main. The mean processed blood volume was 160·6 mL kg^−1^ of donor weight, that is, 9·7 L total. The mean CD34^+^ cell count, cell yield and recipient cell dose from PB were 250·9 × 10^6^ cells, 26·1 × 10^6^ L^−1^ and 4·8 × 10^6^ kg^−1^ of recipient BW, respectively. The mean processed BM volume was 14·3 mL kg^−1^ of donor BW, that is, 0·86 L total. The mean CD34^+^ cell count, cell yield and recipient cell dose from BM before management were 395·7 × 10^6^ cells, 492·6 × 10^6^ L^−1^ and 7·9 × 10^6^ kg^−1^ of recipient BW, respectively. The mean CD34^+^ cell count, cell yield and recipient cell dose from BM after management were 374·9 × 10^6^cells, 468·0 × 10^6^ L^−1^ and 7·5 × 10^6^ kg^−1^ of recipient BW, respectively. The final mean total CD34^+^ cell count, cell yield and recipient cell dose were 625·8 × 10^6^ cells, 10·6 × 10^6^ kg^−1^, and 12·3 × 10^6^ kg^−1^ of recipient BW, respectively. According to our definition of poor yield, eight donors (7·7%) and four donors (3·8%) had a yield <2 × 10^6^ cells kg^−1^ recipient BW after PB and BM collection, respectively. The final product achieved for all donors was > 6 × 10^6^ cells kg^−1^ recipient BW.

### CD34^+^ yield threshold in PB and BM

Aphaeresis and BM collection were performed once in all patients. A total of 60 (57·7%) and 96 (92·3%) donors achieved a CD34^+^ cell dose of 4 × 10^6^ kg^−1^ of recipient BW after aphaeresis procedure and BM collection, respectively. Of the 24 (23·1%) and 44 (42·3%) donors with PB and BM harvest was >6 × 10^6^ kg^−1^, respectively. Although eight donors and four donors attained PB and BM CD34^+^ cell count of <2× 10^6^ kg^−1^ in the one collection, respectively, the final product of all donors reached the number of ≥6 × 10^6^ kg^−1^ of recipient BW (Table 3).

**Table 3:** Donor percentage that met different harvest outcome criteria of CD34^+^ cell count according to recipient BW

	≥4 ×10^6^ kg^−1^ (%)	≥6 ×10^6^ kg^−1^ (%)	≤2 ×10^6^ kg^−1^ (%)
PB	57·7	23·1	7·7
BM	92·3	42·3	3·8
Final product	100	100	0

BM, bone marrow; BW, body weight; PB, peripheral blood.

### Factors affecting PBSC yield

Considering the influence of gender, male donors had significantly higher mean total CD34^+^ cell count (*P* < 0·0001) and cell yield (*P* < 0·0001) compared with female donors. The male donors had higher pre-aphaeresis haemoglobin (HGB) and donor cell dose but lower pre-aphaeresis PLT counts, pre-aphaeresis WBC count and age. Processed volume per kilogram of donor BW, actual flow rate of aphaereses, BMI, G-CSF dose, total processed volume and pre-aphaeresis CIC counts and recipient cell dose did not differ between the genders.

In multiple regression analysis, factors that significantly correlated with CD34^+^ cell yields were donor age, BMI, sex, flow rate and pre-aphaeresis WBC and CIC counts. Age and flow rate were negatively correlated with cell yield, whereas BMI and pre-aphaeresis WBC and CIC counts were positively correlated with cell yield. G-CSF dose and total processed blood volume did not affect the number of CD34^+^ cells kg^−1^ recipient BW.

Regarding the OR of each factor for poor CD34^+^ cell yield (<2 × 10^6^ kg^−1^ recipient BW), female gender and older age were significantly associated with increased risk based on logistic regression analysis. There was a trend of decreased risk for poor yield in donors with higher pre-aphaeresis laboratory data.

### Factors influencing BM stem cells yield

Considering the influence of gender, male donors had significantly higher mean total CD34^+^ cell count (*P* < 0·0001) and cell yield (*P* < 0·0001) before and after management compared with female donors. The male donors had higher pre-BM collection HGB and donor and recipient cell doses before and after management but lower pre-BM collection PLT counts and age. Total processed volume, BMI, G-CSF dose, pre-BM collection WBC and CIC counts did not differ between the sexes.

In multiple regression analysis, factors that significantly correlated with CD34^+^ cell yield were donor age and pre-BM collection CIC counts. Age was negatively correlated with cell yield, whereas pre-BM collection CIC counts were positively correlated with cell yield. G-CSF dose, total processed BM volume and pre-BM collection WBC did not affect the number of CD34^+^ cells L^−1^ of processed BM.

Regarding the OR of each factor for poor CD34^+^ cell yield (<2 × 10^6^ kg^−1^ recipient BW), older age was significantly associated with increased risk based on logistic regression analysis. There was a trend of decreased risk for poor yield in donors with higher pre-BM collection laboratory data.

### Procedure tolerability

All donors tolerated G-CSF administration well. No donor required a reduced or increased G-CSF dosage because of a high leukocyte count on the third day of mobilisation. The most common symptoms during mobilisation were bone pain and myalgia. These were resolved with naproxen.

All donors tolerated the aphaeresis procedure. Symptoms related to hypocalcaemia, such as numbness and muscle cramps, were the most common adverse events; these resolved with calcium gluconate infusion and did not necessitate stopping the procedure. No major morbidity or mortality related to catheterisation was noted. There were no instances of bleeding complications, vascular access problems, or severe thrombocytopaenia during the aphaeresis sessions.

All donors tolerated the procedure of BM collection. There was no bleeding, low blood pressure or other symptoms. No other adverse diathesis was noted.

## DISCUSSION

HLA-haploidentical/mismatched donors offer several advantages: (i) immediate donor availability for virtually all transplant candidates; (ii) ability to select the best of many relatives on the basis of age, infectious disease status and natural killer cell alloreactivity; (iii) controlled graft composition; (iv) immediate access to donor-derived cellular therapies if required after transplantation. Furthermore, for nearly all patients who face graft rejection, HLA-haploidentical/mismatched transplantation offers the advantage of either another family member who is immediately available as an alternative donor or even a second graft from the original donor (Aversa *et al.*, 2008). However, haploidentical/mismatched HSCT with PBSCs or BM alone remains difficult with high GVHD and immune reconstitution (Passweg, 2006). To overcome these problems, the unmanipulated HLA-haploidentical/mismatched related HSCT was performed with combined G-PBSCs and G-BM because of higher volume of collected stem cells and GVHD and GVL regulations (Huang *et al.*, 2004, 2007; Lu *et al.*, 2006; Dong *et al.*, 2007; Chen *et al.*, 2009). Previous studies used different settings for aphaeresis equipment and different protocols, varied type and dosages of G-CSF and various parameters for outcome analysis and reported different final results from unrelated healthy donors (Anderlini *et al.*, 1997; de la Rubia *et al.*, 2002; Diaz *et al.*, 2003; Ikeda *et al.*, 2004; Kozuka *et al.*, 2004; Lysak *et al.*, 2005; Suzuya *et al.*, 2005; Ings *et al.*, 2006; Vasu *et al.*, 2008). However, little information is available about the factors predicting the outcome of PBSC collection and BM harvest from unmanipulated HLA-haploidentical/mismatched related donors.

Final infused cell dose is frequently used as the outcome indicator when analysing the effect of donor characteristics on PBSC harvest. Today, most harvest centres perform at least two aphaereses if the first product does not achieve the target cell dose. The repeated aphaereses may mask the impact of donor factors on stem cell harvest with different numbers of aphaereses among donors and lead to bias due to the differences in criteria for a second aphaeresis among institutions. In addition, most previous studies have adjusted the CD34^+^ cell dose based on donor/recipient BW or on the volume of blood processed to standardise the yields among donors. Consequently, using the final number of CD34^+^ cells collected as the dependent variable may be inappropriate (Vasu *et al.*, 2008). The number of CD34^+^ cells L^−1^ of processed blood volume collected was used as a factor to identify the stem cell yield and this should also be investigated (Sierra *et al.*, 1997). In this study, we comprehensively analysed the factors that affect the stem cell yield with CD34^+^ cells kg^−1^ recipient BW and CD34^+^ cells L^−1^ of processed blood volume collected, which may compensate the deficiency only used alternative methods. Our results showed that the two methods have similar findings for the poor yield, which indicated that any method could be used to analyse the aphaeresis yield for unmanipulated HLA-haploidentical/mismatched related HSCT with combined G-PBSCs and G-BM.

There is a well-documented correlation between PB CD34^+^ count and CD34^+^ cell yield, although not always observed in healthy PBSC donors (Moncada *et al.*, 2003). Other parameters that have been reported to predict CD34^+^ yield of aphaeresis are circulating WBC counts, BMI and the presence of immature myeloid forms (myelocytes, metamyelocytes, etc.) in the PB (Cassens *et al.*, 2004; Kozuka *et al.*, 2004; Suzuya *et al.*, 2005). In our study, we found that the BMI, pre-aphaeresis WBC and CIC counts have significant impact on both CD34^+^ cells kg^−1^ recipient and CD34^+^ cells L^−1^ of processed blood volume collected.

Increasing donor age was associated with a modest negative effect on CD34^+^ mobilisation response (Anderlini *et al.*, 1997). Similar report showed that older age is associated with poor mobilisation and concluded that larger, male donors younger than 55 years would be preferable (Ings *et al.*, 2006). In contrast, other groups have reported that age is not a significant predictive factor (Miflin *et al.*, 1996). In murine studies designed to determine whether aging is associated with changes in stem cell pool size or altered progenitor cell response to cytokines, the study found that aged mice exhibit better mobilisation responses to G-CSF and that their haematopoietic progenitor cells were characterised by reduced adhesion to marrow stroma (Xing *et al.*, 2006). In our study, the results showed that age was negatively correlated with CD34^+^ yields.

Blood cell separators separate the different blood components on the basis of their individual densities by centrifugation. Stem cells are found within the buffy coat, which consists of the WBCs and platelets, as well as in the top of the RBC layer. The buffy coat is detected by the optic device. When the buffy coat is detected, valves are opened to transport the PBSCs into the storage bag (Moog, 2004). The relationship between the flow rate and the results of stem cell harvest is reported by few authors. They showed that a lower blood withdrawal rate was associated with a higher MNC yield and CD34^+^ cell yield (Suzuya *et al.*, 2005; Wang *et al.*, 2008). Our study also indicates that the yield of cells correlated negatively with flow rate. The cause for the results may be that entire time was utilized with low flow rate to separate the blood components and to detect the buffy coat by the optic device. However, the flow rate was not clearly defined or even described in previous studies and in our studies of factors affecting stem cell collection outcome. An optimal flow rate may need to be confirmed in a prospective setting.

The PB CD34^+^ cell count in healthy subjects under unstimulated conditions is very low, but it increases 15- to 35-fold following 4–5 days of G-CSF administration (Rhodes & Anderlini, 2008). The outcome of stem cell harvest depends on two steps: mobilisation and harvest. But few have described procedural factors affecting the outcome of PBSC collection. Some recent studies have focused on the effect of large-volume leukaphaeresis (LVL) (Wang *et al.*, 2008). Although LVL may indeed increase final stem cell yield, other possible procedural factors, such as flow rate and circulation access, may also increase yield. In this study, we found that the dosage of twice daily G-CSF administration and the total processed blood volume did not affect the CD34^+^ yield.

PLT counts 1 day prior to or on the day of stem cell harvest were associated with CD34^+^ harvest (Suzuya *et al.*, 2005; Tomblyn *et al.*, 2005). The association of higher baseline PLT counts with improved CD34^+^ mobilisation may be related to common pathways of thrombopoiesis and progenitor cell mobility. Increased plasma levels of stromal-derived factor-1 have been shown to enhance human thrombopoiesis and mobilise human colony forming cells in non-obese diabetic/severe combined immunodeficiency mice (Perez *et al.*, 2004). In addition, CD34^+^ cells may exhibit changes similar to platelet-derived microparticles during G-CSF mobilisation (Nomura *et al.*, 2004). However, the CD34^+^ yields were not associated with the pre-aphaeresis PLT counts in this study.

The effect of gender on stem cell yield in our study disagreed with those of previous studies (Engelhardt *et al.*, 1999; de la Rubia *et al.*, 2002; Kozuka *et al.*, 2004; Lysak *et al.*, 2005). Two recent large studies found higher total final CD34^+^ cell count and cell yield in male donors (Fischer *et al.*, 2005; Ings *et al.*, 2006). The significantly lower numbers of post-G-CSF circulating CD34^+^ cells in female donors were detected by another study (Vasu *et al.*, 2008). We noted that female gender had a lower mean harvest outcome and a higher risk for poor yield in our cohort. The actual underlying reasons for the relatively poor mobilisation by G-CSF in female donors remain to be elucidated. The development of newer mobilising agents, such as CXCR4 antagonists, should be explored with regard to the CD34^+^ cell mobilisation in female donors.

The final adequacy of the product is determined by the CD34^+^ cell count of the PBSC aphaeresis product. While the collection target is usually 4–5 × 10^6^ kg^−1^ recipient BW, the minimum number of CD34^+^ cells required to ensure prompt engraftment after allogeneic transplantation is probably in the range of 2 × 10^6^ kg^−1^ recipient BW (Rhodes & Anderlini, 2008). A small number of donors (up to 5%) prove to be poor mobilisers and fail to reach even the minimum CD34^+^ cell dose despite two or more collections (Anderlini *et al.*, 1999). Previous studies have shown delayed engraftment at CD34^+^ doses less than 8 × 10^6^ kg^−1^ BW (Koh & Chao, 2008). Most physicians would usually target for ‘megadose’ of stem cell (>10 × 10^6^ CD34^+^ cells kg^−1^ recipient BW) from the donor while planning for HLA-haploidentical transplants, and this can place considerable demand on both the donors and the phaeresis service for the following reasons: (i) The high graft contents are easily achieved in children but can be a major obstacle in adults. (ii) The immobility from long hours of phaeresis can be exhausting for the donors and the procedure is often associated with significant aphaeresis-related adverse effects. (iii) For the aphaeresis and stem cell processing laboratory staff, the procedures involved can be time-consuming and labour-intensive (Lang *et al.*, 2004). The BM harvest was performed in our study and was found that age was negatively correlated with CD34^+^ yield, whereas pre-BM collection CIC counts were positively correlated with CD34^+^ yield, and G-CSF dose, total processed BM volume and pre-BM collection WBC did not affect the CD34^+^ yield.

Most common side effects are mild-to-moderate, with bone pain, myalgia, malaise and nausea being the most frequently occurring during mobilisation (Stroncek *et al.*, 1996). These side effects are readily treated with common analgesics, such as acetaminophen. G-CSF also causes a mild-to-moderate decrease in PLT count from baseline in approximately 25% of healthy allogeneic donors (Tassi *et al.*, 2005). The aphaeresis procedure itself causes an additional and more substantial PLT depletion and transient G-CSF-induced hypercoagulability in PBSC donors (Falanga *et al.*, 1999; Topcuoglu *et al.*, 2004). On the other hand, the coagulation system may change because of the use of large anti-coagulation drugs. Hence bleeding appears to be common; however, no bleeding was observed in our study. In this study, 1–2 units of donor's own blood were prepared and infused back into the donor during BM harvest because a significant amount of blood was taken with the marrow, which prevented low blood pressure during harvest and minimised the need for a blood transfusion from an outside source after the harvest and stopped the side effect of allotransfusion.

In conclusion, we found that female gender, older age and higher flow rate were significantly associated with increased risk for poor harvest of PB. A higher BMI may decrease the risk. Age was negatively correlated with CD34^+^ cell yield of BM, whereas pre-BM collection CIC counts were positively correlated with CD34^+^ cell yield. Therefore in a donor with multiple high-risk characteristics (e.g. an older female with a lower BMI) for poor CD34^+^ cell yield, strategies for increasing CD34^+^ cell yield must be considered besides repeated aphaeresis. BM stem cells could be an alternative source in these donors because our data showed no correlation between gender and CICs of PB in BM harvest.

## References

[b1] Anderlini P, Donato M, Chan KW, Huh YO, Gee AP, Lauppe MJ, Champlin RE, Korbling M (1999). Allogeneic blood progenitor cell collection in normal donors after mobilization with filgrastim: the M.D. Anderson Cancer Center experience.. Transfusion.

[b2] Anderlini P, Przepiorka D, Seong C, Smith TL, Huh YO, Lauppe J, Champlin R, Korbling M (1997). Factors affecting mobilization of CD34^+^ cells in normal donors treated with filgrastim. Transfusion.

[b3] Aversa F, Reisner Y, Martelli MF (2008). The haploidentical option for high-risk haematological malignancies. Blood Cells, Molecules, and Diseases.

[b4] Ball LM, Bernardo ME, Roelofs H, Lankester A, Cometa A, Egeler RM, Locatelli F, Fibbe WE (2007). Cotransplantation of ex vivo-expanded mesenchymal stem cells accelerates lymphocyte recovery and may reduce the risk of graft failure in haploidentical hematopoietic stem-cell transplantation. Blood.

[b5] Cassens U, Barth I, Baumann C, Fischer RJ, Kienast J, Vormoor J, Sibrowski W (2004). Factors affecting the efficacy of peripheral blood progenitor cells collections by large-volume leukapheresis with standardized processing volumes. Transfusion.

[b6] Chen X, Zhang C, Zhang X (2009). Role of antithymocyte globulin and granulocyte-colony stimulating factor-mobilized bone marrow in allogeneic transplantation for patients with hematologic malignancies. Biology of Blood and Marrow Transplantation.

[b7] de la Rubia J, Arbona C, de Arriba F (2002). Analysis of factors associated with low peripheral blood progenitor cell collection in normal donors. Transfusion.

[b8] Diaz MA, Sevilla J, de la Rubia J (2003). Factors predicting peripheral blood progenitor cell collection from pediatric donors for allogeneic transplantation. Haematologica.

[b9] Dong L, Wu T, Zhang MJ, Gao ZY, Lu DP (2007). CD3^+^ cell dose and disease status are important factors determining clinical outcomes in patients undergoing unmanipulated haploidentical blood and marrow transplantation after conditioning including antithymocyte globulin. Biology of Blood and Marrow Transplantation.

[b10] Engelhardt M, Bertz H, Afting M, Waller CF, Finke J (1999). High-versus standard dose filgrastim (rhG-CSF) for mobilization of peripheral blood progenitor cells from allogeneic donors and CD34(+) immunoselection. Journal of Clinical Oncology.

[b11] Falanga A, Marchetti M, Evangelista V (1999). Neutrophil activation and hemostatic changes in healthy donors receiving granulocyte colony stimulating factor. Blood.

[b12] Fischer JC, Frick M, Wassmuth R, Alexander P, Michael P, Peter W (2005). Superior mobilisation of haematopoietic progenitor cells with glycosylated G-CSF in male but not female unrelated stem cell donors. British Journal of Haematology.

[b13] Huang XJ, Chang YJ, Zhao XY (2007). Maintaining hyporesponsiveness and polarization potential of T cells after in vitro mixture of G-CSF mobilized peripheral blood grafts and G-CSF primed bone marrow grafts in different proportions. Transplantation Immunology.

[b14] Huang XJ, Han W, Xu LP (2004). A novel approach to human leukocyte antigen-mismatched transplantation in patients with malignant hematological disease. Chinese Medical Journal.

[b15] Ikeda K, Kozuka T, Harada M (2004). Factors for PBPC collection efficiency and collection predictors. Transfusion and Apheresis Science.

[b16] Ings SJ, Balsa C, Leverett D, Mackinnon S, Linch DC, Watts MJ (2006). Peripheral blood stem cell yield in 400 normal donors mobilised with granulocyte colony-stimulating factor (G-CSF): impact of age, sex, donor weight and type of G-CSF used. British Journal of Haematology.

[b17] Koh LP, Chao NJ (2008). Nonmyeloablative allogeneic hematopoietic stem cell transplant using mismatched/haploidentical donors: a review. Blood Cells, Molecules, and Diseases.

[b18] Kozuka T, Ikeda K, Teshima T (2004). Peripheral blood circulating immature cell counts predict CD34^+^ cell yields in G-CSF-induced PBPC mobilization in healthy donors. Transfusion.

[b19] Lang P, Bader P, Schumm M (2004). Transplantation of a combination of CD133^+^ and CD34^+^ selected progenitor cells from alternative donors. British Journal of Haematology.

[b20] Lu DP, Dong LJ, Wu T (2006). Conditioning including antithymocyte HLA-mismatched/haploidentical blood and marrow transplantation can achieve comparable outcomes with HLA identical sibling transplantation. Blood.

[b21] Lysak D, Koza V, Jindra P (2005). Factors affecting PBSC mobilization and collection in healthy donors. Transfusion and Apheresis Science.

[b22] Miflin G, Charley C, Stainer C, Anderson S, Hunter A, Russell N, Russell N (1996). Stem cell mobilization in normal donors for allogeneic transplantation: analysis of safety and factors affecting efficacy. British Journal of Haematology.

[b23] Moncada V, Bolan C, Yau Y, Leitman SF (2003). Analysis of PBPC cell yields during large-volume leukapheresis of subjects with a poor mobilization response to filgrastim. Transfusion.

[b24] Moog R (2004). Apheresis techniques for collection of peripheral blood progenitor cells. Transfusion and Apheresis Science.

[b25] Nomura S, Inami N, Kanazawa S, Iwasaka T, Fukuhara S (2004). Elevation of platelet activation markers and chemokines during peripheral blood stem cell harvest with G-CSF. Stem Cells.

[b26] Passweg JR (2006). Natural-killer-cell-based treatment in haematopoietic stem-cell transplantation. Best Practice and Research: Clinical Haematology.

[b27] Perez LE, Alpdogan O, Shieh JH (2004). Increased plasma levels of stromal-derived factor-1 (SDF-1/CXCL12) enhance human thrombopoiesis and mobilize human colony-forming cells (CFC) in NOD/SCID mice. Experimental Hematology.

[b28] Rhodes B, Anderlini P (2008). Allogeneic peripheral blood stem cell collection as of 2008. Transfusion and Apheresis Science.

[b29] Schattenberg AV, Dolstra H (2005). Cellular adoptive immunotherapy after allogeneic stem cell transplantation. Current Opinion in Oncology.

[b30] Sierra J, Storer B, Hansen JA (1997). Transplantation of marrow cells from unrelated donors for treatment of high risk acute leukemia: the effect of leukemic burden, donor HLA-matching, and marrow cell dose. Blood.

[b31] Sorror ML, Maris MB, Storer B, Scandmaier BM, Diaconescu R, Flowers C, Maloney DG, Storb R (2004). Comparing morbidity and mortality of HLA-matched unrelated donor hematopoietic cell transplantation after nonmyeloablative and myeloablative conditioning: influence of pretransplantation comorbidities. Blood.

[b32] Stroncek DF, Clay ME, Petzoldt ML, Smith J, Jaszcz W, Oldham FB, McCullough J (1996). Treatment of normal individuals with granulocyte-colony-stimulating factor. Donor experiences and the effects on peripheral blood CD34^+^ cell counts and on the collection of peripheral blood stem cells.. Transfusion.

[b33] Suzuya H, Watanabe T, Nakagawa R (2005). Factors associated with granulocyte colony-stimulating factor-induced peripheral blood stem cell yield in healthy donors. Vox Sanguinis.

[b34] Tassi C, Tazzari PL, Bonifazi F (2005). Short-and long-term haematological surveillance of healthy donors of allogeneic peripheral haematopoietic progenitors mobilized with G-CSF: a single institution prospective study. Bone Marrow Transplantation.

[b35] Tatsumi K, Otani H, Sato D, Enoki C, Iwasaka T (2008). Granulocyte-colony stimulating factor increases donor mesenchymal stem cells in bone marrow and their mobilization into peripheral circulation but does not repair dystrophic heart after bone marrow transplantation. Circulation Journal.

[b36] Tomblyn M, Gordon L, Singhal S, Tallman MS, Williams S, Winter JN, Evens A, Mehta J (2005). Use of total leukocyte and platelet counts to guide stem cell apheresis in healthy allogeneic donors treated with G-CSF. Bone Marrow Transplantation.

[b37] Topcuoglu P, Arat M, Dalva K, Ozcan M (2004). Administration of granulocyte-colony-stimulating factor for allogeneic hematopoietic cell collection may induce the tissue factor dependent pathway in healthy donors. Bone Marrow Transplantation.

[b38] Vasu S, Leitman SF, Tisdale JF (2008). Donor demographic and laboratory predictors of allogeneic peripheral blood stem cell mobilization in an ethnically diverse population. Blood.

[b39] Wang TF, Wen SH, Chen RL (2008). Factors associated with peripheral blood stem cell yield in volunteer donors mobilized with granulocyte colony-stimulating factors: the impact of donor characteristics and procedural settings. Biology of Blood and Marrow Transplantation.

[b40] Xing Z, Ryan MA, Daria D (2006). Increased hematopoietic stem cell mobilization in aged mice. Blood.

[b41] Zhang C, Chen X, Zhang X, Gao L, Kong PY, Wang QY, Peng X, Liu H (2008). Mobilization of peripheral blood stem cells for autologous transplantation patients with hematologic malignancies: influence of disease, mobilization method, age and sex. Transfusion and Apheresis Science.

